# Comparison of routine versus targeted HIV testing strategies: coverage and estimated missed infections in emergency room and primary care centre

**DOI:** 10.7448/IAS.17.4.19671

**Published:** 2014-11-02

**Authors:** María Jesús Pérez Elías, Cristina Gomez-Ayerbe, Alfonso Muriel, Maria Eugenia Calonge, Alberto Diaz, Pilar Pérez Elías, Maria Martinez-Colubi, Almudena Uranga, Cristina Santos, Ana Moreno, Carmen Quereda, Enrique Navas, Santiago Moreno

**Affiliations:** 1 Infectious Diseases, Hospital Ramón y Cajal, IRYCIS, Madrid, Spain; 2 Statistics Department, Hospital Ramón y Cajal, IRYCIS, CIBERESP, Madrid, Spain; 3 Family Care, Centro de Salud García Noblejas, Madrid, Spain; 4 Internal Medicine, Hospital de Sanchinarro, Madrid, Spain

## Abstract

**Introduction:**

Different HIV Testing Strategies (TS) and clinical care settings had not been face to face evaluated [1]. We compared coverage, Newly Diagnosed HIV Infection (NDHI) and Estimated Missing HIV Infections (MHI) in Hospital Emergency Room (HER) and Primary Care Center (PCC), in DRIVE study (Spanish acronym of HIV infection Rapid Diagnosis) and in clinical practice the year before DRIVE.

**Materials and Methods:**

In DRIVE study, 18–60 years old, non-HIV-infected population visiting an HER or a PCC were proposed both a structured risk practices and clinical conditions questionnaire (RP&CC-Q) and a rapid HIV test. This arm is the HIV Routine TS. We analyze a hypothetical arm, where risk practices were universally assessed with an RP&CC-Q, subsequently risk-positive patients where HIV tested, Targeted-TS. Coverage was assessed as the ratio of tested population (TP)/attended population (AP) in HER and PCC. TP/AP ratios were also calculated in the year before, the Clinical Practice-TS. NDHI was expressed per ‰ tests performed. MHI was estimated assuming in the non-tested population, overall DRIVE rate of NDHI ‰ and NDHI ‰ in negative RP&CC-Q.

**Results:**

A total of 5329 RP&CC-Q and rapid HIV tests were performed to 49.64% women, median age 37 (28–47) years old, mainly 74.9% Spaniards. Confirmed NDHI was 4.1‰, and in 48, 8% of RP&CC-Q negative NDH was 0‰. HIV screening coverage was always better in PCC than in HER, and higher in DRIVE study than in clinical practice. Estimated MHI was higher in HER and in the clinical practice-TS. Targeted-TS coverage was lower, but resulted in similar NDHI and MHI than routine-TS, testing half the population, see Table 1.

**Conclusions:**

Best HIV Testing Strategy is routine-TS in Primary Care Center. Targeted-TS resulted in same newly HIV diagnoses and missed HIV infections than routine-TS with half the resources employed.

**Table 1:** Coverage, newly diagnosed HIV infections, missed HIV infections

Settings	Hospital emergency room	Hospital emergency room	Primary Care Center	Primary Care Center
Testing strategies	Coverage	NDI‰/no MHI	Coverage	NDI‰/no MHI
Clinical practice 2011/2012	0.31	0/139	3.7	2.4/23
DRIVE routine 2012/2013	2.6	8.6/128	32.9	2.2/16
DRIVE targeted 2012/2013	1.43	8.6/128	16.25	2.2/16
